# Involvement of Small Colony Variant-Related Heme Biosynthesis Genes in *Staphylococcus aureus* Persister Formation *in vitro*

**DOI:** 10.3389/fmicb.2021.756809

**Published:** 2021-12-23

**Authors:** Xuyang Wang, Weizheng Li, Wenjie Wang, Shiyong Wang, Tao Xu, Jiazhen Chen, Wenhong Zhang

**Affiliations:** ^1^Department of Infectious Diseases, National Medical Center for Infectious Diseases, Shanghai Key Laboratory of Infectious Diseases and Biosafety Emergency Response, Huashan Hospital, Shanghai Medical College, Fudan University, Shanghai, China; ^2^Department of Infectious Diseases, Putuo Hospital, Shanghai University of Traditional Chinese Medicine, Shanghai, China; ^3^National Clinical Research Center for Aging and Medicine, Huashan Hospital, Fudan University, Shanghai, China; ^4^Key Laboratory of Medical Molecular Virology (MOE/MOH) Shanghai Medical College, Fudan University, Shanghai, China

**Keywords:** heme, *Staphylococcus aureus*, persister, stress response, small colony variants

## Abstract

**Background:** Persisters are important reasons for persistent infections, and they can lead to antibiotic treatment failure in patients and consequently chronic infection. *Staphylococcus aureus* small colony variants (SCVs) have been shown to be related to persistent infection. Mutations in the genes of the heme biosynthesis pathway lead to the formation of SCVs. However, the relationship between heme production genes and persister has not been tested.

**Methods:***HemA* and *hemB* were knocked out by allelic replacement from *S. aureus* strain USA500 separately, and then, the heme deficiency was complemented by overexpression of related genes and the addition of hemin. The stress-related persister assay was conducted. RNA-sequencing was performed to find genes and pathways involved in heme-related persister formation, and relative genes and operons were further knocked out and overexpressed to confirm their role in each process.

**Results:** We found that heme biosynthesis deficiency can lead to decreased persister. After complementing the corresponding genes or hemin, the persister levels could be restored. RNA-seq on knockout strains showed that various metabolic pathways were influenced, such as energy metabolism, amino acid metabolism, carbohydrate metabolism, and membrane transport. Overexpression of *epiF* and operon *asp23* could restore USA500∆*hemA* persister formation under acid stress. Knocking out operon *arc* in USA500∆*hemA* could further reduce USA500∆*hemA* persister formation under acid and oxidative stress.

**Conclusion:** Heme synthesis has a role in *S. aureus* persister formation.

## Introduction

Persisters, which were first described in *Staphylococcus aureus* in 1944 (Bigger, 1944), are of major clinical concern worldwide. Persisters are important causes of persistent infections, including endocarditis, dermatitis, and meningitis (Fisher et al., 2017), and are well described in *Escherichia coli*, *Pseudomonas aeruginosa*, *Mycobacterium tuberculosis*, *Salmonella enterica* subsp. *enterica* serovar Typhimurium, and *Staphylococcus aureus* (Helaine and Kugelberg, 2014; Harms et al., 2016; Michiels et al., 2016). The presence of persisters can lead to antibiotic treatment failure in patients (Mechler et al., 2015; Van den Bergh et al., 2016) and may facilitate evolution toward resistance (Cohen et al., 2013; Levin-Reisman et al., 2017).

*Staphylococcus aureus*, as a commensal pathogen, can cause multiple infections in humans, including cellulitis, endocarditis, osteomyelitis, bacteremia, and septic shock (Harms et al., 2016; Michiels et al., 2016). When its three type II toxin–antitoxins are knocked out, persister formation is not decreased in both the exponential and stationary phases (Conlon et al., 2016). In the exponential phase, a reduction in the ATP level induces persister formation, and in the stationary phase, persister formation is associated with a low membrane potential (Wang et al., 2018). These findings provide a link between persisters and respiratory-deficient small colony variants (SCVs).

SCVs have been found to be involved in persistent and recurrent infections (Kahl et al., 2016). In addition, mutations in genes encoding enzymes involved in electron transport can lead to the formation of respiratory-deficient SCVs (Proctor, 2019). For example, a deficiency in heme biosynthesis can lead to SCV formation. However, the relationship between deficiency in heme biosynthesis and persister formation remains unclear.

Previously, we screened a transposon mutagenesis library of *S. aureus* USA500 under antibiotic pressure (Wang et al., 2015) and identified two *hemA* insertion mutants. The *hemA* and *hemB* genes encode the first- and third-step enzymes for heme biosynthesis, respectively (Choby et al., 2018), the deficiency of which can lead to heme deficiency. In this study, we investigated the possible relationship between heme synthesis and persister formation in *S. aureus*. This study elucidates the important role of SCV formation in the mechanism of persistent staphylococcal infections to find potential treatment targets.

## Materials and Methods

### Bacterial Strains and Reagents

*Staphylococcus aureus* USA500 was used as the wild-type strain throughout the study. All strains used in this study are described in Table 1. The plasmid, pmx16, was constructed for continuous expression of exogenous genes. Briefly, primers pmx16-f and pmx16-r (Supplementary Table S1) were mixed, cooled from 72°C to 4°C, and ligated to one segment. The segment with multiple restriction sites was digested with *Kpn*I and *Sac*I (New England Biolabs, United States) and inserted into pCM29 (Pang et al., 2010) to replace the sGFP-encoding gene.

**Table 1:** Bacterial strains used in this study.

Name of strains	Details
USA500	Type strain
USA500∆hem*A*	*hemA* knockout mutant in USA500
USA500∆*hemB*	*hemB* knockout mutant in USA500
USA500∆hemA::pRB473-*hemAX*	*hemAX* complemented by overexpression through recombining with plasmid pRB473 in *hemA* knockout mutant in USA500
USA500∆*hemB*::pRB473-*hemB*	*hemB* complement by overexpression through recombining with plasmid pRB473 in *hemB* knockout mutant in USA500
USA500∆*hemA*::pmx16-*1,606*	operon *USA300HOU1605, 1,606, 1,607, 1,608, 1,609* overexpressed through recombining with plasmid pmx16 in USA500∆*hemA*
USA500∆*hemA*::pmx16-*hut*	operon *hutIU* overexpressed through recombining with plasmid pmx16 in USA500∆*hemA*
USA500∆*hemA*::pmx16-*asp*	operon *asp23 -USA300HOU2176, 2,177, 2,178* overexpressed through recombining with plasmid pmx16 in USA500∆*hemA*
USA500∆*hemA*::pmx16-*epiF*	gene epiF overexpressed through recombining with plasmid pmx16 in USA500∆*hemA*
USA500∆*hemA*∆*als*	operon *alsSD* was further knocked out from USA500∆*hemA*
USA500∆*hemA*∆*arc*	operon *arcA3B3D3C3-USA300HOU2631* was further knocked out from USA500∆*hemA*
USA500∆*hemA*::pmx16	plasmid pmx16 was electric transformed into USA500∆*hemA*

### Growth Curves

All strains were grown to the stationary phase, then diluted to 1:1,000 in 5 ml of TSB medium, and incubated at 37°C and 220 rpm. Among them, USA500∆*hemA*, USA500∆*hemB* was diluted to 7:1000 in 5 ml of TSB medium. Colony-forming units (CFU) were counted for the USA500, USA500∆*hemA*, USA500∆*hemB*, USA500∆*hemA*::pRB473-*hemAX*, and USA500∆*hemB*::pRB473-*hemB* strains at 0, 3, 6, 10, 16, 24, 36, and 48 h by plating 10 μl aliquots of 10-fold serial dilutions on TSA in triplicate. For the hemin complementation experiment, all strains were grown to the stationary phase, then diluted to 1:1,000 in 5 ml of TSB medium, and incubated at 37°C and 220 rpm. Hemin was added at a concentration of 5 μg/ml from the beginning of growth. All experiments were repeated three times.

### Persister Assay

All strains were diluted to 1:100 in the stationary phase in a fresh TSB. Because of their slight growth deficiency, the USA500∆*hemA*, USA500∆*hemB*, USA500∆*hemA*::pRB473-*hemAX*, and USA500∆*hemB*::pRB473-*hemB* mutants were cultured for 28 h, while USA500 was cultured for 24 h so that all of them reached an equivalent stationary phase. The final bacterial concentration was adjusted to the same level (approximately 1 × 10^9^ CFU/ml) to minimize the quorum sensing influence on the persister assay. After centrifugation, 1 × 10^9^ CFU of the stationary phase bacteria were resuspended in 1 ml of TSB and incubated under different stress conditions, including heat (57°C, 3 h), acid (pH 3.0, 48 h), oxidative stress (50 mM H_2_O_2_; 3 h), and ciprofloxacin (400 μg/ml, 7 days). Aliquots were withdrawn from each treatment at different time points, and live bacteria were counted as described above. For the hemin complementation experiment, hemin was added at a concentration of 5 μg/ml from the beginning of growth before all kinds of treatment. The bacterial load detection limit was 10^1^ CFU. All the experiments were replicated at least three times. Because USA500∆*hemA* and its derivatives were very sensitive to all stresses, to compare their differences, the heat and oxidative stress conditions were changed to 55°C and 10 mM H_2_O_2_, respectively.

### Identification and Verification of the Deficiency of Transposon Mutants Under Levofloxacin Exposure

The previously constructed transposon mutant library was further screened for mutants vulnerable to levofloxacin (12.5 μg/ml) under the same conditions (Li et al., 2009; Wang et al., 2015). The transposon mutants were identified using two rounds of inverse PCR, as described previously (Wang et al., 2015).

To confirm their decreased survival phenotype, *hemA* insertion mutants were treated with levofloxacin (12.5 μg/ml) in TSB medium for 3–6 days at 37°C. The replica was transferred to Tryptone Soy Agar (TSA) plates and compared with the wild-type strain.

### Construction of Knockout Mutants and Complementation Strains

An allelic replacement method was used to knock out *hemA* and *hemB*, as described previously (Bae and Schneewind, 2006; Wang et al., 2015; Xu et al., 2017). For *hemA*, the upstream and downstream allelic sequences were amplified with the following primers: *hemA*-1-KpnI, *hemAX*-1Rev, *hemAX*-2Rev, and *hemAX*-2-EagI (Supplementary Table S1). Subsequently, the two PCR fragments were ligated using overlapping PCR. The allelic fragment was cut by *Kpn*I and *Eag*I and inserted into the plasmid PKOR2 to construct the plasmid PKOR2-*hemA*. For *hemB*, the *arc* and *alsSD* operons were knocked out, and gene-1-*Kpn*I/*Nco*I-1, gene-1 Rev./gene-2 Rev., and gene-2-*Eag*I/*Eco*RI-2 were used as primers (Supplementary Table S1).

After the candidate genes were knocked out, a shuttle plasmid, pRB473, was used to construct complementation strains. For *hemAX* and *hemB* complementation, the genes were amplified with the primers listed in Supplementary Table S1 and ligated to the plasmid, pRB473. For *hemB* complementation, *hemB*-Pro pstI-1F and Hem-promoter-1R were used as primers to amplify the promoter, which was then ligated to the *hemB* part. To complement certain genes in *S. aureus* USA500∆*hemA*, the plasmid, pmx16, was used. For overexpression of *epiF* and the *hutIU*, *USA300HOU1605–09*, and *asp23* operons, gene-F and gene-R were used as primers for amplification. The primers used are listed in Supplementary Table S1. The recombinant plasmids were transformed into the target strain by electroporation.

### RNA Isolation and RNA-Seq

The strains of USA500, namely USA500∆*hemA*, USA500∆*hemB*, USA500∆*hemA*::pRB473-*hemAX*, and USA500∆*hemB*::pRB473-*hemB*, were cultured until all of them reached an equivalent stationary phase. They were then treated with levofloxacin (25 mg/ml, 48 h). Total RNA was isolated from these five strains using the RNeasy mini kit (Qiagen, United States) according to the manufacturer’s protocol. For library construction, rRNA was depleted from the total RNA extracted, followed by RNA fragmentation. After first- and second-strand cDNA synthesis, end repair, adding poly(A) tails, adapter ligation, PCR, and product purification, the library was successfully constructed. For quality analysis, SOAP (Li et al., 2008) was used to screen the RNA-seq data, and HISAT (Kim et al., 2015) was used to align the clean reads to the USA300 TCH1516 genome sequence. Rockhopper (Tjaden, 2015) was used to reconstruct the transcripts and classify them into the intergenic region, sense, partially overlapping, and antisense transcripts. Finally, Bowtie2 (Langmead and Salzberg, 2012) was used to align reads to known and novel mRNAs, both of which were regarded as references. The expression levels were calculated using the RSEM software.

### Real-Time PCR

Three replicate total RNA samples were separately extracted from strains USA500, USA500∆*hemA*, USA500∆*hemB*, USA500∆*hemA*::pRB473-*hemAX*, and USA500∆*hemB*::pRB473-*hemB* using the RNeasy mini kit (Qiagen) and reverse transcribed using the PrimeScript RT reagent kit (TaKaRa, Japan). Real-time PCR was performed using SYBR Premix Ex Taq II (TaKaRa, Japan) with the primers listed in Supplementary Table S2 to compare expression levels of 38 selected genes. All the kits were used according to the manufacturer’s instructions. The real-time PCR conditions were as follows: 95°C for 30 s, followed by 40 cycles of 95°C for 5 s, 60°C for 30 s, and 95°C for 15 s and then 60°C for 1 min, 95°C for 15 s, and cooling at 4°C. The gene expression levels were calculated using the 2^−∆∆Ct^ method (Livak and Schmittgen, 2001) with *pta* and *hu* (Valihrach and Demnerova, 2012) as the housekeeping genes.

### Statistical Analysis

Statistical analysis was performed using the one-way ANOVA with Dunnett’s *post hoc* test in multiple strains in the GraphPad Prism 8 software (GraphPad Software, San Diego, CA, United States). For *in vitro* experiments, data from three independent experiments were pooled. *p* < 0.05 was considered statistically significant. The PossionDis algorithm was used to screen for differentially expressed genes using the RNA-seq data.

## Results

### Characterization of *hemA* and *hemB* Knockout Mutants of *S. aureus*

**Figure 1:**
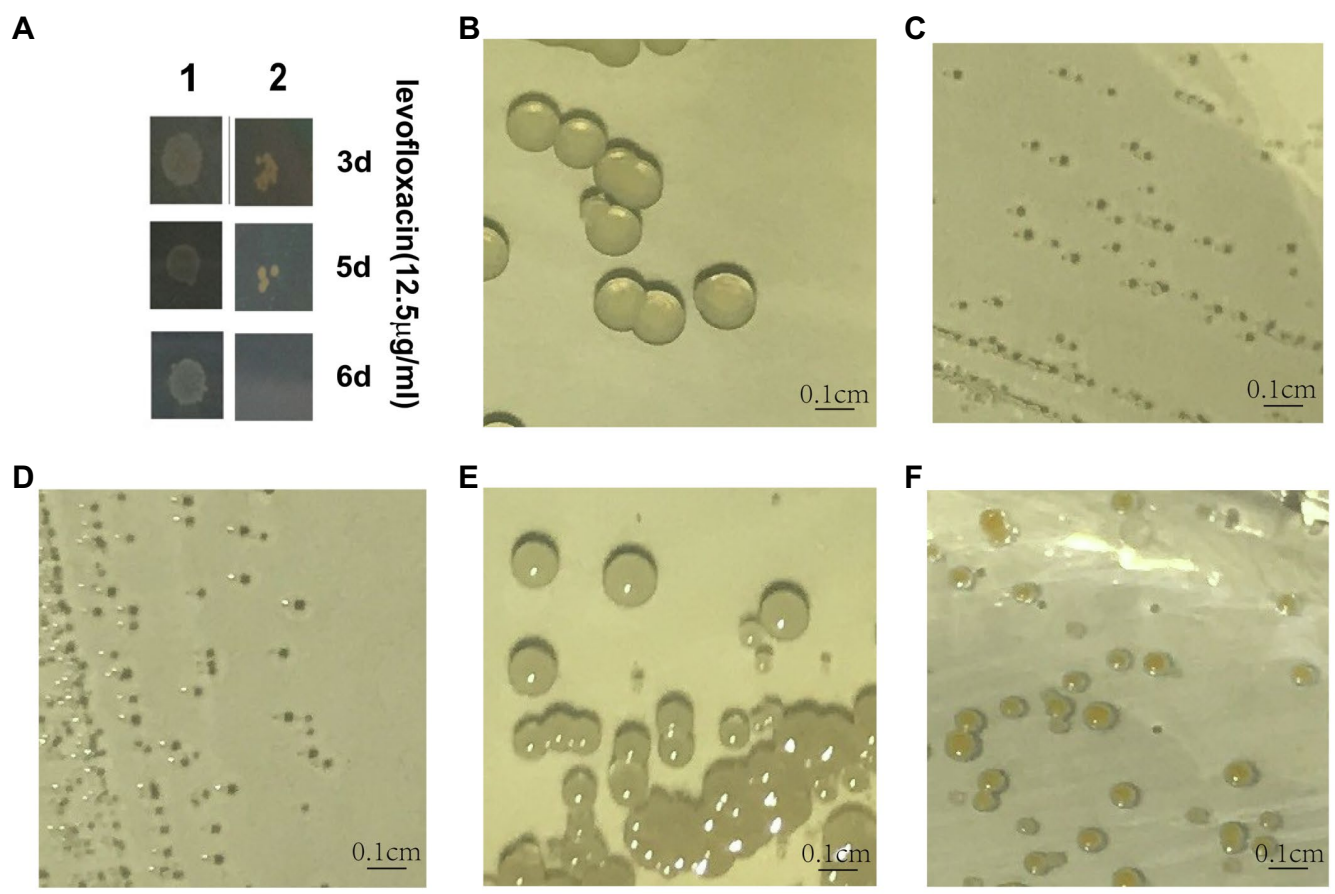
Characterization of *hemA* and *hemB* knockout mutants of *Staphylococcus aureus*. **(A)** “1” stands for negative control; “2” stands for USA500*hemA* mutant; and 3, 5, and 6 days indicate CFU counts after 3, 5, and 6 days of treatment with levofloxacin. **(B–F)** Indicate the colonies of USA500, USA500∆*hemA*, USA500∆*hemB*, USA500∆*hemA*::pRB473-*hemAX*, and USA500∆*hemB*::pRB473-*hemB*, respectively, after growth at 37°C for 24 h. The short black dash in panel A indicates a length of 0.1 cm.

In addition to the 260 clones that were found to have decreased survival after 6 days of levofloxacin exposure in the previous study (Wang et al., 2015), we identified seven clones with deficiency in persister formation that had a transposon insertion between nucleotides 854 and 855 of *hemA* (Figure 1A; Supplementary Figure S1). And mutant in *hemA* resulting in SCV has been reported before (Curtis et al., 2016; Hubbard et al., 2021).

**Figure 2:**
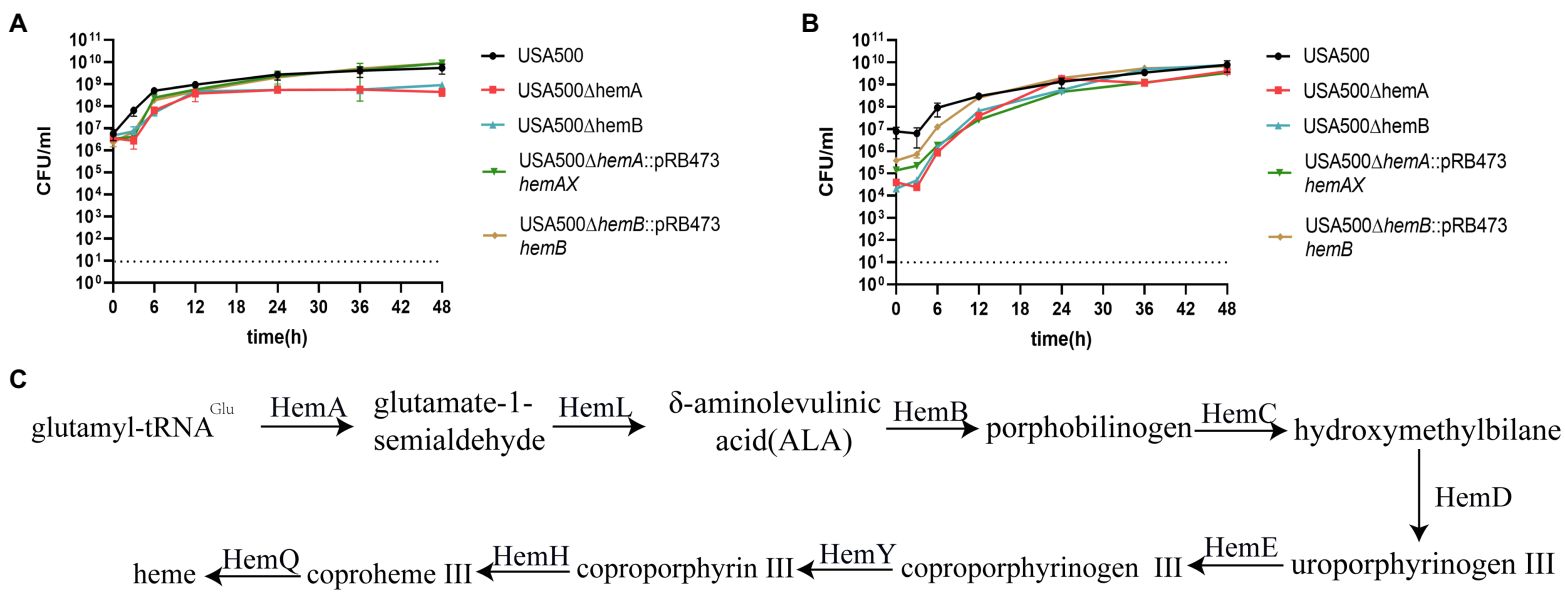
Heme biosynthesis deficiency inhibits the growth of *Staphylococcus aureus*. **(A)** The growth curves of USA500 ∆*hemA* and ∆*hemB* mutants. **(B)** Hemin supplemented at the concentration of 5 μg/ml can restore the growth of USA500 ∆*hemA* and ∆*hemB* mutants. The data shown are the average of three biological replicates with the error bar shown. **(C)** The overview of the pathway of heme biosynthesis in *S. aureus*.

To examine whether the heme biosynthesis pathway is associated with persister formation, we knocked out *hemA* and the downstream *hemB* gene by allelic gene replacement from *S. aureus* USA500 wildtype (Figure 1B). When either of these two genes was knocked out, the strains displayed a typical SCV phenotype (Figures 1C,D; Jonsson et al., 2003; Garcia et al., 2012; Proctor et al., 2014; Hubbard et al., 2021). After complementing the knockout genes separately, the phenotype was both reverted and the reversion was complete (Figures 1E,F). After culturing on TSA plates for 24 h, the colonies of the mutants were only 0.1 mm in diameter compared with those of the wild-type strain, which had a diameter of 2 mm and were lighter yellow in color. In addition, the growth rates of both ∆*hemA* and ∆*hemB* strains were lower (Figure 2A). After 24 h, the CFU counts for the ∆*hemA* and ∆*hemB* strains were both 0.68 log lower than that for USA500. After culturing for 48 h, the stationary-phase bacterial concentrations of USA500∆*hemA* and USA500∆*hemB* were approximately seven times lower than that of the wild type.

When the heme genes were complemented or hemin was added to the culture medium at a concentration of 5 μg/ml, the growth deficiency and the SCV phenotype in the mutant strains were reversed and the same phenomenon had been reported before (Balwit et al., 1994; Batko et al., 2021), which demonstrated that the growth deficiency was due to the lack of heme biosynthesis (Figure 2B). *hemA* and *hemB* encode the first and third enzyme in the biosynthesis process of heme synthesis (Figure 2C), and our results showed knockout either of the two genes impair the production of heme and therefore cause growth deficiency.

### Involvement of Heme in Persister Formation Under Stress Conditions

**Figure 3:**
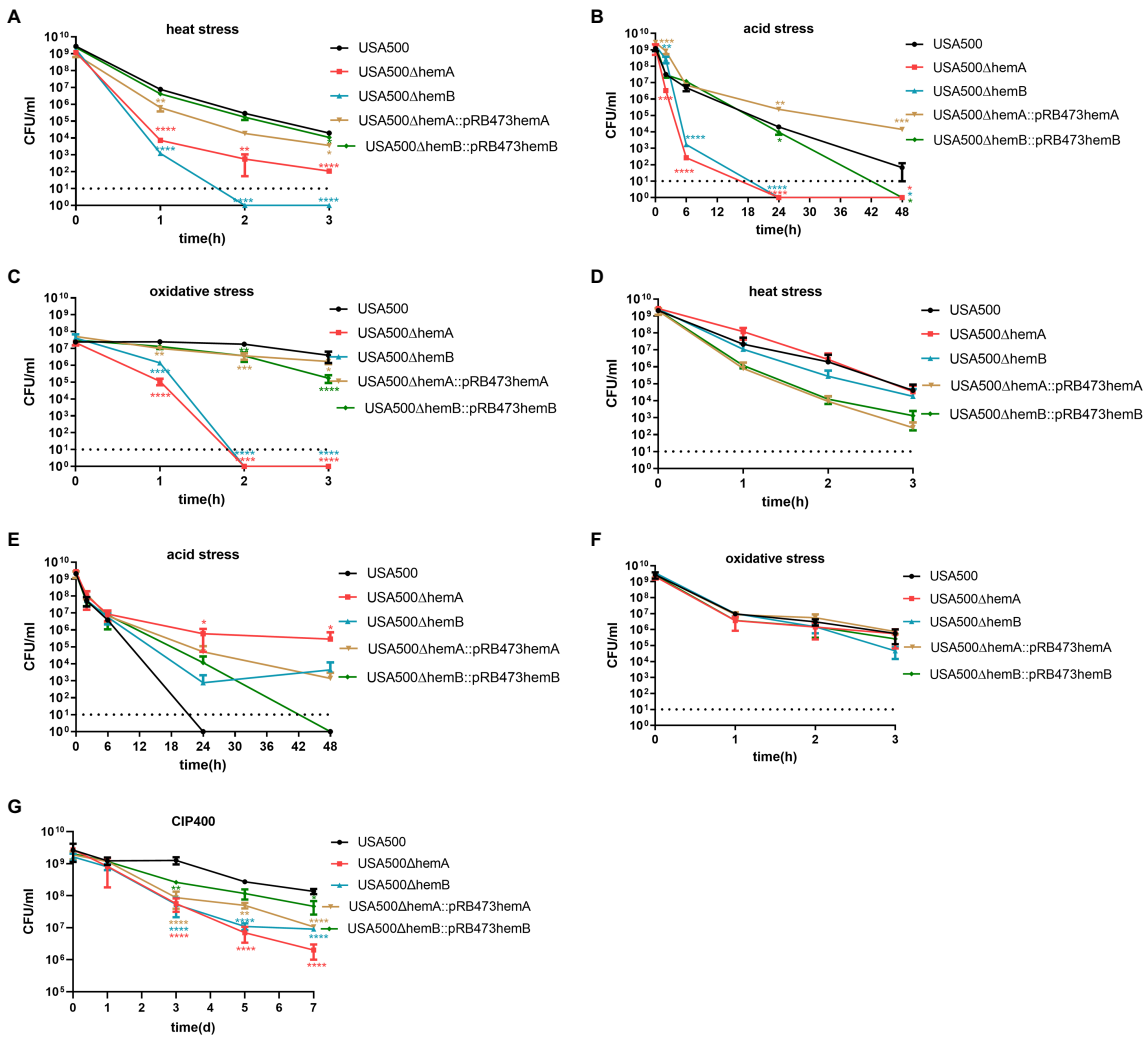
Heme biosynthesis deficiency impacts persister formation. **(A,C,E,G)** Demonstrate that *hemA* mutant and *hemB* mutant persister formation was inhibited under heat stress (57°C), acid stress (pH = 3), oxidative stress (H_2_O_2_; 50 mM), and antibiotic stress (ciprofloxacin; 400 μg/ml). The *hemA* and *hemB* gene complements can restore the persister formation phenotype. **(B,D,F)** Demonstrate hemin supplementation at a concentration of 5 μg/ml can mitigate the inhibition of persister formation under heat stress (57°C), acid stress (pH = 3), and oxidative stress (H_2_O_2_; 50 mM) for *hemA* and *hemB* mutants. The data shown are the average of three or more biological replicates with an error bar. Statistical significance was determined with one-way ANOVA test and Dunnett’s multiple comparisons test to compare a mutant strain with the wild type; *p < 0.05; **p < 0.01; ***p < 0.001; ****p < 0.0001.

Persister formation by these SCV strains was tested under stress conditions, including heat, acidity, and oxidative stress. Under heat stress, the persister levels were lower in the ∆*hemA* and ∆*hemB* mutants than in the wild-type strain, by 3.0 log (*p* < 0.0001) and 3.8 log (*p* < 0.0001) after 1 h, respectively (Figure 3A). The numbers of persister cells in the ∆*hemA* and ∆*hemB* mutants were reduced to 0 after 2 and 3 h, respectively. When the corresponding genes were complemented in the ∆*hemA* and ∆*hemB* mutants, the persister levels were restored to the equivalent level in the wild-type strain (*p* > 0.05; Figure 3A).

Under inorganic acid stress, the persister levels in the ∆*hemA* and ∆*hemB* mutants were 4.2 and 3.4 log lower, respectively, than those in the wild-type strain after 6 h exposure. Consistently, both ∆*hemA* and ∆*hemB* mutants were killed after 24 h exposure (Figure 3B). Similarly, under oxidative stress, the persister levels in the ∆*hemA* and ∆*hemB* mutants were 2.4 log (*p* < 0.0001) and 1.2 log (p < 0.0001) lower than those in the wild-type strain after 1 h, respectively, and both mutants were eliminated after 2 h (Figure 3C). The overexpressing strains, ∆*hemA*::pRB473-*hemAX* and ∆*hemB*::pRB473-*hemB*, showed that their persister levels were restored under both oxidative and inorganic acid stress conditions. Not only complementing the heme biosynthesis genes could restore persistence of the mutants under stress conditions but adding hemin (5 μg/ml) to the medium could also rescue persister levels in both ∆*hemA* and ∆*hemB* strains to almost those in the wild-type strain under heat, inorganic acid stress, and oxidative conditions (Figures 3D–F).

When the cells were treated with ciprofloxacin at the concentration of 400 mg/ml (Figure 3G), after 5 days, persister level of ∆*hemA* and ∆*hemB* was 1.59-log(*p* < 0.001) and 1.40-log(*p* < 0.01) lower than the wild-type strain and ∆*hemA*::pRB473-*hemAX* and ∆*hemB*::pRB473-*hemB* were 0.74-log (p < 0.01) and 0.37-log lower (*p* > 0.05). After 7 days, persister levels of ∆*hemA* and ∆*hemB* were 1.83-log (p < 0.001) and 1.18-log (p < 0.01) lower than the wild-type strain and ∆*hemA*::pRB473-*hemAX* and ∆*hemB*::pRB473-*hemB* were 1.11-log (p < 0.01) and 0.47-log (p > 0.05) lower. These results showed that persister level of ∆*hemA* and ∆*hemB* mutants was reduced under ciprofloxacin stress and complementing the respective genes may not always restore the phenotype.

The data indicate that heme was involved in persister formation in *S. aureus*, and the lack of heme biosynthesis could result in severe deficiency in persister formation under various stress conditions.

### Related Genes and Pathways in Heme Biosynthesis-Deficient Strains

**Figure 4:**
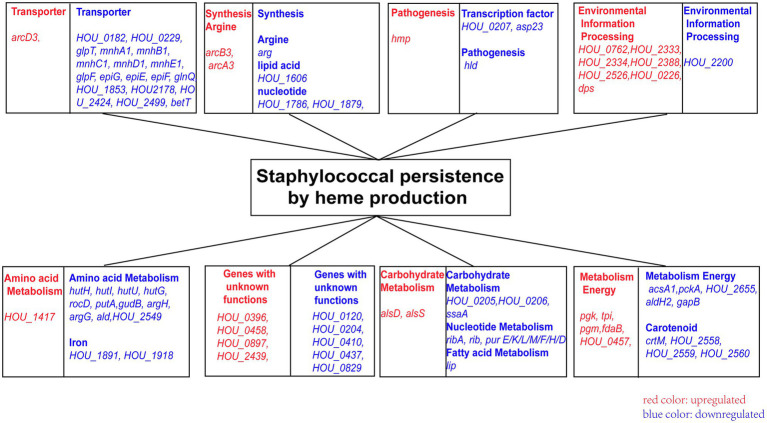
The pathway and genes associated with heme deficiency. Blue indicates genes downregulated, and red indicates genes upregulated.

RNA-seq analysis was conducted in the ∆*hemA* and ∆*hemB* strains to investigate the downstream genes and pathways after the heme biosynthesis pathway was disrupted, which could provide a clue for the deficiency of persister formation. A total of 64 genes were downregulated, and 23 genes were upregulated in both ∆*hemA* and ∆*hemB* mutants (Supplementary Tables S3 and S4). The downregulated genes were mainly associated with amino acid metabolism (*hutI, hutU, hutG*, and *hutH*), nucleotide metabolism (*purE, purK, purL, purF, purM, purH*, and *purD*), carbohydrate metabolism (*acsA1*), transporters (*mnhA1, mnhB1, mnhC1, mnhD1, mnhE1, glpF, glnQ*, and *glpT*), lipid metabolism (*lip*), carotenoid biosynthesis (*crtM*), and environmental information processing (*epiF, epiE, epiG, hld*, and *asp23*). Some of these differentially expressed genes were clustered in the same operons (Figure 4).

The upregulated genes were associated with energy metabolism (*pgk, tpi, pgm*, and *fdaB*), butanoate metabolism (*alsS* and *alsD*), arginine biosynthesis (a*rcD3, arcB3*, and *arcA3*), transporters (*USA300HOU_0762* and *USA300HOU_0897*), and a multidrug efflux pump (*USA300HOU_2333* and *USA300HOU_2334*).

The RNA-seq results were further confirmed by real-time PCR using the wild type as a control (Supplementary Table S5). Among 34 genes that were selected, transcriptional changes were confirmed in 21 genes, indicating that a large part of the RNA-seq results was reliable.

### Genes Involved in Heme Biosynthesis and Metabolism Influence Persister Formation

Since some operons were entirely upregulated or downregulated, they were more likely to be consequent downstream genes or complementary genes of heme deficiency. We therefore constructed strains overexpressing four downregulated genes or operons in USA500∆*hemA*, including the *hutIU* (involved in Histidine metabolism), *USA300HOU1605–09*, and *asp23* (encoding alkaline shock protein 23 and other protein) operons and *epiF* (involved in Quorum sensing), and constructed two double-knockout strains, including ∆*arc* (encoding arginine/ornithine antiporter) and ∆*alsSD* (involved in Butanoate metabolism) in USA500∆*hemA*, to further investigate the mechanism of heme effects on persister formation.

**Figure 5:**
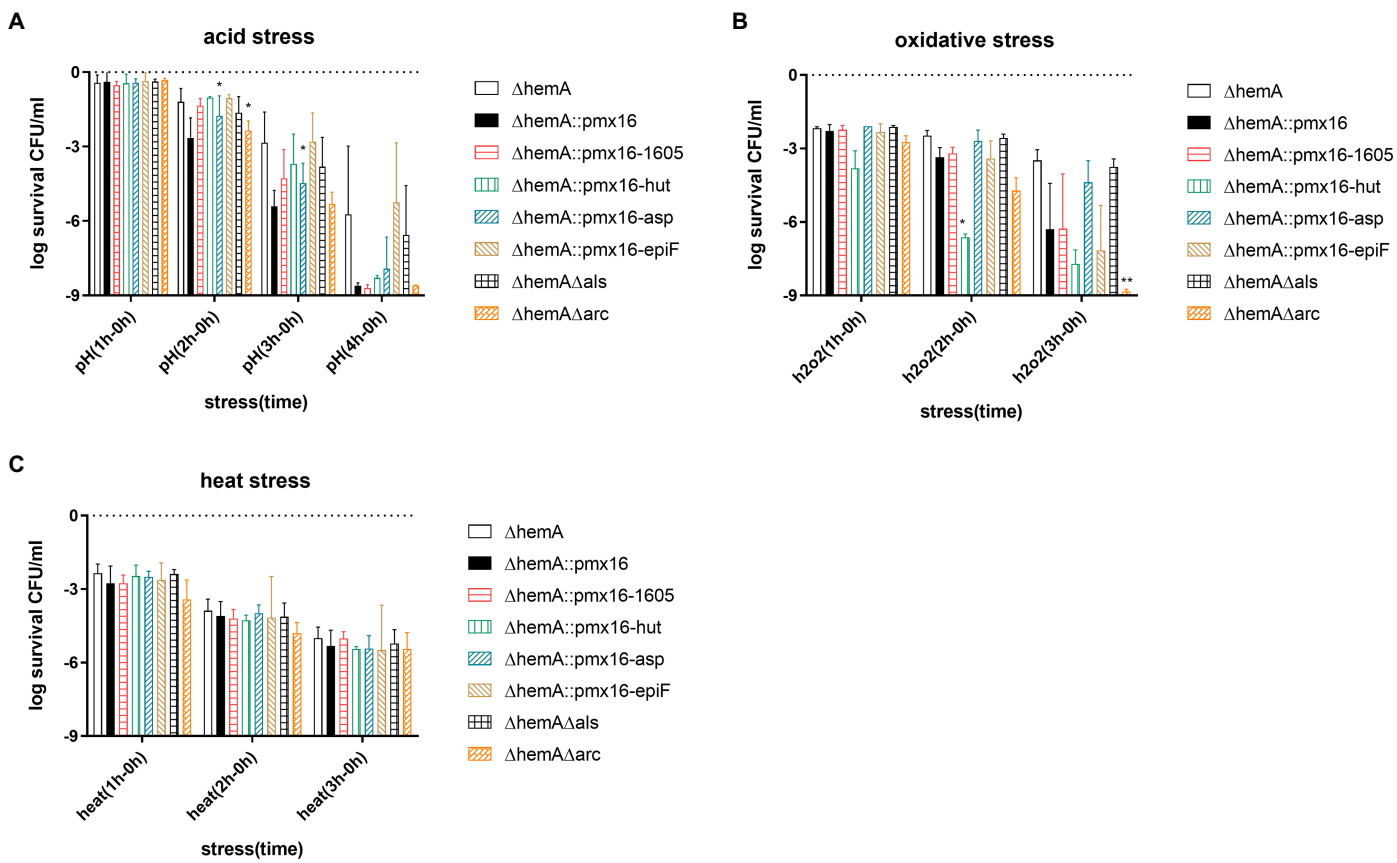
Genes associated with heme biosynthesis deficiency. **(A–C)** Demonstrate *hemA* mutant persister formation level under acid stress (pH = 3), oxidative stress (H_2_O_2_; 10 mM), and heat stress (55°C). Operon *hutIU*, operon *USA300HOU1605,1,606,1,607,1,608,1,609*, operon *asp23-USA300HOU2176,2,177,2,178*, and gene *epiF* were overexpressed in USA500∆*hemA*. Operon *arcA3B3D3C3-USA300HOU2631* and *alsSD* were knocked out further from USA500∆*hemA*. The data shown are an average of three biological replicates with an error bar. Statistical significance was determined using one-way ANOVA test and Dunnett’s multiple comparisons test to compare a mutant strain with the control strain. The USA500∆*hemA* was the control for the double knockout strain, and USA500∆*hemA*::pmx16 was the control for overexpressing strain. *p < 0.05; **p < 0.01.

Under acid stress, the overexpression of the asp23 operon restored persister formation in the ∆*hemA* mutant, with 0.56 and 0.93 log higher survival than that in the ∆*hemA* control after 2 h and 3 h treatment, respectively (*p* < 0.05). However, the double ∆*hemA*∆*arc* mutants had 1.16log fewer CFU than that in the USA500∆*hemA* mutant after 2 h treatment (*p* < 0.05; Figure 5A), suggesting that knocking out the arc operons further inhibited persister formation under acid stress. Under oxidative stress, the overexpression of the *hutIU* operon and the knockout arc operon further decreased persister formation. The USA500 ∆*hemA*::pmx16-*hutIU* strain had 3.27 log fewer CFU than those in the ∆*hemA* control after 2 h treatment (*p* < 0.05). The double USA500∆*hemA*∆*arc* mutant had 5.35 log fewer CFU than those in the control after 3 h treatment (*p* < 0.05; Figure 5B). No significant changes were observed under heat stress. (*p* > 0.05; Figure 5C).

## Discussion

*Staphylococcus aureu*s SCVs are commonly found in recurrent infections. Among bacteria, SCVs may have some advantages over the wild-type strains. The formation of SCVs has been reported to be a beneficial survival strategy for bacteria to persist in sites of infection (Proctor et al., 2006) as this feature can promote internalization and protect bacteria from being eliminated. According to Tuchscherr et al. (2010), SCVs may be able to persist inside the human body because they elicit little inflammatory response and cytotoxicity, which provides a survival advantage over wild-type bacteria.

Heme biosynthesis is important for the physiology of most bacteria, as heme is integral in the electron transport chain. Therefore, heme deficiency can reduce the transmembrane potential and impair the transport of cationic antimicrobials, such as aminoglycosides and antifolate antibiotics, which affects the treatment outcome (Garcia et al., 2013). The heme-dependent QoxABCD or CydAB terminal oxidase is indispensable in the last step of respiration, which is the reduction of oxygen to water, in all bacteria (Hammer et al., 2013, 2016). Different hemoproteins, including catalase and bacterial nitric oxide synthase, play important roles in bacterial pathogenesis and growth (Cosgrove et al., 2007; van Sorge et al., 2013; Mogen et al., 2017). When the ∆*hemA* and ∆*hemB* mutants were cultured to perform the persister assay, the growth rates and the stational phase CFU counts were lower than those of the wild-type strain. Considering the effect of quorum sensing (Xu et al., 2017) on the formation of persister cells, we increased the CFU number of these two mutants at the beginning of any treatment, with a 7-fold increase in cell density, according to the growth curves. Consequently, the data showed that the difference in the persister formation was due to heme deficiency rather than to the effect of quorum sensing.

Heme deficiency is a known reason for SCV formation, and the loss of heme genes is common in *S. aureu*s, especially under stress (Proctor et al., 2006). However, the survival advantage of SCVs only applies to *in vivo* conditions. Based on the study of Painter et al. (2017), the lack of heme can result in a deficiency in the electron transport chain and cause bacterial resistance to oxidative burst in coculture with whole human blood. When heme was complemented, the susceptibility to oxidative stress was restored, which suggests that heme complementation might promote the clearance of persister cells. Similarly, a recent study has shown that ∆*hemB* SCVs can promote glycolysis to induce necroptosis, which can reduce the eradication of SCVs and thus increase bacterial pathogenicity (Wong Fok Lung et al., 2020). Normally SCV is innately tolerant of antibiotics but not relative with resistance genes (Edwards, 2012). The decrease in the electrochemical potential in the cell wall of SCV can lower the entry of aminoglycosides and thus increase survival (Melter and Radojevič, 2010). In addition, the lower metabolism level of SCV can influence the effect the antibiotics which rely on the active growth of bacteria (Garcia et al., 2013). However, the more adaptable to *in vivo* condition of SCV do not seem to be adaptive *in vitro*. In our study, under multiple stress conditions, the persistence of SCV strains significantly decreased, which seems unreasonable for this way of adaption. We speculated that *in vivo* conditions were quite different from *in vitro* conditions and the reasons of difference need to be further illustrated.

In our study, *hemA* and *hemB* mutants showed downregulation of various *pur* genes, some of which (such as *purB* and *purM* mutants) showed defective persistence under various conditions, such as acid, antibiotics, and heat stress (Yee et al., 2015). The mRNA expression of the *asp23* operon decreased after levofloxacin treatment in our study. Among the genes, *asp23* has been reported to be associated with daptomycin resistance and tolerance (Barros et al., 2019). Similarly, in our research, when *asp23* was overexpressed, the persister level was restored under acid stress. It seems that heme deficiency may be complemented by Asp23, which is one of the most abundant cytosolic proteins in stationary *S. aureus* cells, with a copy number of over 25,000 molecules per cell (Maass et al., 2011). However, the concrete mechanism still needs to be elucidated. *USA300HOU_2,178*, in the same operon with *asp23*, is predicted to encode a glycine betaine transporter, named OpuD, which is necessary for glycine betaine uptake and osmoprotection in *E. coli* (Kappes et al., 1996).

The *arc* operon participates in arginine biosynthesis, with *arcD3* encoding an arginine/ornithine antiporter. When four genes of this operon were knocked out in our study, the antioxidative stress ability of the mutants was further impaired, implying the importance of the *arc* operon in the persister formation mechanism. This operon allows USA300, which is involved in the development of skin and soft tissue infections, to thrive in an acidic environment (Thurlow et al., 2013). However, in our study, when this operon was knocked out in heme-deficient mutant strains, persister formation was not significantly disrupted under acid stress. This effect may have been caused by a redundancy of arginine biosynthesis pathways or a sufficient free arginine level in the culture medium. Nevertheless, under oxidative stress, this redundancy could not alleviate the decrease in persister formation, which implies that different mechanisms may be involved in persister formation under acid and oxidative stress conditions, thus warranting further study.

The *hutI* and *hutU* genes participate in histidine metabolism, and *hutU* is regulated by the Agr system according to a previous study (Xu et al., 2017). In our study, the overexpression of *hutI* and *hutU* in heme-deficient mutants could restore persister formation under oxidative stress, which suggests that the damaged heme metabolism can result in the destruction of histidine metabolism and then cause a decrease in persister formation. This hypothesis can be verified by complementing histidine in the culture in future studies.

This study had some limitations. We only tested the role of heme deficiency in persister formation *in vitro* but not in animal experiments, which could have further elucidated the *in vivo* infection mechanism of heme-deficient strains. Second, we only explored the persistence phenotype of strain USA500. The possibility of strain differences should be explored further in more clinical strains. Third, the RNA-seq experiment performed in stress conditions may be better to illustrate the difference between the knockout bacteria. However, after stress exposure, as shown in Figure 3, the cells were killed quickly. We have difficulties that the RNA extracted after stress exposure was merely mRNA fractures of lysis cells, not from the live persister cells. The RNA of persister cells was not enough to perform RNA-seq. In addition, as the cells were killed quickly, it can be inferred that gene expression could be very unstable and varies over time, and it is difficult to perform biological replications in very short time. Furthermore, as the cells were killed so quickly, like oxidative and heat stress, that the differences in phenotype can hardly be attributed to the change of transcriptomics after stress, it could be partially due to the difference in the original expression profile of the bacteria before stress. Therefore, the difference in the expression profile in non-stress condition can in some way determine the survival phenotype of the bacteria under stress conditions. In our study, we chose to use stationary phase bacteria after culturing for 48 h to compare the transcriptome.

In summary, our research demonstrates that heme deficiency results in the impairment of growth and persister formation in *S. aureus*. This provides a link between SCV formation and persisters and provides clues for finding potential treatment targets for persistent *S. aureus* infections.

### Data Availability Statement

The RNA-sequencing data was uploaded to the SRA database under the Bioproject ID of PRJNA750238.

## Author Contributions

XW and WL performed most of the experiments. SW helped with the construction of the strains. WW provided the key target genes associated with the persister formation. JC and XW wrote the essay. TX helped with the revision process. JC and WZ designed the experiments and helped to review the essay. All authors contributed to the article and approved the submitted version.

## Funding

This research was sponsored by the National Natural Science Fund (Grant No. 81471987). This work was also supported by Research grants from the Shanghai Science and Technology Committee (20dz2260100, 20Z11901100, and 20dz2210400) and Key Discipline Construction Plan from Shanghai Municipal Health Commission (GWV-10.1-XK01).

## Conflict of Interest

The authors declare that the research was conducted in the absence of any commercial or financial relationships that could be construed as a potential conflict of interest.

## Publisher’s Note

All claims expressed in this article are solely those of the authors and do not necessarily represent those of their affiliated organizations, or those of the publisher, the editors and the reviewers. Any product that may be evaluated in this article, or claim that may be made by its manufacturer, is not guaranteed or endorsed by the publisher.

## Supplementary Material

Supplementary Figure S1 Transposon insertion site in *hemA*. This figure indicates screened clones with Tn917 transposon insertion between nucleotides 854 and 855 of *hemA*.
