# Supplementary material for: Involvement of Small Colony Variant-Related Heme Biosynthesis Genes in Staphylococcus aureus Persister Formation in vitro
Source: Front Microbiol. 2021 Dec 23;12:756809. doi: 10.3389/fmicb.2021.756809 (PMC8733728; doi:10.3389/fmicb.2021.756809)
Supplement: Supplementary file 1 [file table_1.docx]

Table S1 primers for strains construction

| primers | 5’ to 3’ |
| --- | --- |
| hemA-1-KpnI | ATATGGTACC GCGCGTGGTTTACGTTCAATCAT |
| hemAX-1Rev | TAGATTATTTTCATATACTAACAATACAAATATTATAACATAATTTAAAATCCAATTAAGTA |
| hemAX-2Rev | TTGTTAGTATATGAAAATAATCTAGAGCAATCCGTTCCTAG |
| hemAX-2-EagI | TTATCGGCCGTGCTCTTTTAATTTGTTACTCACTGTTTTGC |
| hemB-1-KpnI | ATATGGTACC GATAGAGATACATTGTTACCTGCAATCGG |
| hemB-1Rev | AAATATAAAACCTTTTAGCCCCTACTTTCTAAAATCTTTTCAAT |
| hemB-2Rev | TAGGGGCTAAAAGGTTTTATATTTATGATTTTCCATAAACTGTAGGAG |
| hemB-2-EagI | TTATCGGCCG CGTGTGTGCCGTAGATAAGAATGTAC |
| newhemAPro-  HindIII | ATTCAAGCTTGTACGACAAAACTTATACAAAAATTTTAA  AAATAATGTAAGCA |
| hemAX-Pro-  BamhI-R | TAATGGATCCTCAATTCACAAAATGTGTTGCAAAAAATAAATTAATC |
| hemB-Pro pstI-1F | ATTCCTGCAGGTACGACAAAACTTATACAAAAATTTTAAAAATAATGTAAGCA |
| Hem-promoter-1R | CTATGTCTATCAAATTTCATATATACTAACAATACAAATAT  TATAACATAATTTAAAATCCAATTAAGTACG |
| hemB-2F | TTTGTATTGTTAGTATATATGAAATTTGATAGACATAGAAGATTGAGATCATCA |
| hemB-Pro-  BamhI-2R | TAATGGATCCTTATTTATCTAAATAGCGACAAATGTCCTTTGC |
| EcoRI-epiF Rev | ATATGAATTCTCATTTGTAATCACCTTTCGTTATTTTGAAGAATATCTCTT |
| KpnI-epiF For | ATATGGTACCATGGCAAAATTAGTTACTGAAAACATTTCGAAGC |
| MluI-HutU For | ATATACGCGTTGTAAGCGTTTTAAATTTAGTGGAATTAACTTCTTAAAAGAAC |
| SacI-hutI Rev | ATATGAGCTCGTTTAAACCAATGCTTAGAGCTTTCTAATTATTTTATGCTT |
| MluI-hou_1609 For | ATATACGCGTAGGTATTCAATACTGGTTTATTGCAATACAAAGGG |
| SacI-hou_1605 Rev | ATATGAGCTCACTTTCTTTTTTAAGCTTTAAATTTCTCCCCATTTTTTTAGC |
| MluI-hou_2178 For | ATATACGCGTTTATGGGTATGAACTTACCATTACACAGAAAGGG |
| SacI-asp23 Rev | ATATGAGCTCGCCCTTTTTATCGAATAACTTATTGTAAACCTTGTCT |
| arc-1 Rev | TTACATTGTATAGTCTATTTCCTCCTTTTATCTTTGAATTCATTTAGAGTATAGACA |
| arc-2- EcoRI Rev | ATATGAATTCGGTCAAATGTAGTCTTTTCTAACTTGAAATTACTTGCC |
| arc-1- NcoI For | ATATCCATGGTTTGTAATTAGATGAGAGGAGTGTGAGGGTTGT |
| arc-2 For | GGAGGAAATAGACTATACAATGTAAAATGAATAAGTTGAACATGAGGTCTAACG |
| als-1 Rev | ATATTTCTGGATTCATTATATTCATTTCCCTTCAAATGTGATGTATAGATGTGTATT |
| als-2- EcoRI Rev | ATATGAATTCAATTTATCTATAACTTAAATACACTATAAGTCTCAATTAAAAGTATAAGA |
| als-1- KpnI For | ATATGGTACCTGCGAAATTCTAAAATTTTCTGAACGTAAAAAAACAAAT |
| als-2 For | GAAATGAATATAATGAATCCAGAAATATAATGACGGTTTATGAAAATTGACT |
| pmx16-f | GGTACCAGATCTACGCGTGAGCTCGAATTCGTAATCATGGTCATAGCT |
| pmx16-r | GAGCTCACGCGTAGATCTGGTACCGATGCATCTTGCTCGATACAT |

Table S2 primer sequence for realtime PCR

| Primer | sequence(5' to 3') |
| --- | --- |
| RS00890-F | TCGAAGCGATAATGGCACAGGG |
| RS00890-R | TTGGCAAGCCTTTATGCAAAGGAC |
| RS01120-F | CGAATCCACCTACAGGCGCAG |
| RS01120-R | ACACCAGGCCAATACATCACAGTT |
| RS02390-F | GGCTTTGTAATGGCGCTTGGC |
| RS02390-R | ATTTCGCAGACTCACGTGGCA |
| RS03440-F | CCTAGAGCAATGGCTGCACCT |
| RS03440-R | TTGGTGTTGAGCATATCCGCCA |
| RS03445-F | AATCCTGGTGGTGGGTTTATTGGT |
| RS03445-R | GACCTCCGCTAAGCGACAACA |
| RS03455-F | TGCGTTTGAATGACATGGGAGATAATCG |
| RS03455-R | TCGAATTAATGCATAGGCCCCTACTTTG |
| RS04045-F | GCGGCATGCGGTAACAATTCTG |
| RS04045-R | AATGACTTACCGCCTTCCCCTTTAG |
| RS04350-F | AACAGAGAGTGGCGCAAATGATACT |
| RS04350-R | AACGCGTTTGTCACGGTCTGT |
| RS04885-F | AGCCAACAGCAGCGTCTTGAT |
| RS04885-R | AGGCACCTGCTGCAGAAGTTATT |
| RS05440-F | ATTTGCGGTTCTTGTTTTTCCAGGTTC |
| RS05440-R | ACTGGCTTACCTTCAGCTGCAA |
| RS05445-F | TCGAACCAAGCGTTGAAGAAATTAAACTTG |
| RS05445-R | CTTCACCAGGCCCCATAAGCA |
| RS05450-F | AACATCGTGGTCAAGAAGGTGCA |
| RS05450-R | TCAGAAGACGAATGGAAGATAGCACCT |
| RS05455-F | GGCTTGCGTCAAGTGGCATTC |
| RS05455-R | CATATCCGGCTGGCAATGCAC |
| RS09065-F | CCATCCATTTATCGCGCCTGCT |
| RS09065-R | AGCGTCAGTTTTTGAACACGCAATTG |
| RS09075-F | GAATCGACTCGCACATTAAAGCCTTGA |
| RS09075-R | ACGTGATTTGGTTGCAGCACAAC |
| RS10215-F | ATTTCTTTGCTGATATTTTAGCTCACCCTCA |
| RS10215-R | GAAACAAAGACTTGGAATTGCAATGGCA |
| RS12145-F | TCATCGCCAGCATTACCAGCA |
| RS12145-R | CCACTGGTATGTGAAAGGGCCTAAC |
| RS12420-F | TTTACGGAAGATTTTTGGTGCTGATTCAC |
| RS12420-R | TGGTATCGCTGCACGTGAAGT |
| RS12435-F | TGACCACGGAAAATGGTAACGCA |
| RS12435-R | CAGCAACTTTTGCGACGCTACA |
| RS12545-F | ATCAACGGTCGGAGCTGCATT |
| RS12545-R | GTTGTAACACCACGTTGAGAAGCTAATTC |
| RS12550-F | GAAATCCTGCTGATCCGATACCATGG |
| RS12550-R | TCTTCAGAAGCAGTTTTTGCAGAAATTAAGG |
| RS12555-F | AGCCCTCTTTAATTGCCGCTTCT |
| RS12555-R | CGCAAGTTGTGTCCGTTGCTG |
| RS13210-F | GCAGCATTGACCGTAACAGCATTC |
| RS13210-R | TCCTCTTACCTGCAACGACGTTC |
| RS13215-F | GCAACACCCATACCTGGGTCA |
| RS13215-R | GATGGTAGTGACGCGGTTGGT |
| RS13320-F | CAAGTCGTCCCGGTAACGCAT |
| RS13320-R | GGGTCTACTAGGACCATTTGCAGG |
| RS13325-F | GCTTGCAGCAGTTGCTTTTCCAA |
| RS13325-R | AGGTCAAGATGGCGAAACGAAAGAT |
| RS13375-F | AAGGCATCGTTACCGGCATCA |
| RS13375-R | ATCAAGCAGAGCAAGCACCGT |
| RS13485-F | GTGTTAGCAACGTCAAAAGTTGGTGT |
| RS13485-R | TTGTCGCTTCCATCTCAACGAAACTAC |
| RS13615-F | CGCTCGACAGAATTTAAAGGCACTTATGA |
| RS13615-R | TTCCCACGTTCTAGATCTGCAATTGATTG |
| RS13805-F | AATTGGGGGCGCAATTGGAAC |
| RS13805-R | CCAGCCAATGACAAAGCCACC |
| RS14525-F | TCTTCGCTTCTCGCGCGTTTA |
| RS14525-R | GAAGATGTTTGGACACCGGAGGATAT |
| RS14535-F | ACGAGACGTAACCACACCAGC |
| RS14535-R | GCAGCGCCATCTTCAAATGGT |
| RS14735-F | ACGATATGCGTACACGTGTGGTAAC |
| RS14735-R | TGACGTTCGTTTGCACGGTCT |
| RS14920-F | AGTTGCTTGCTGTCGATGCTCTAA |
| RS14920-R | GGATGGACACTGCTTGCTGGT |
| RS14925-F | TCCACACCGGTACACCAGAGA |
| RS14925-R | AATTCGACGAGAACGCGTGCT |
| RS14930-F | TCCATTGCTCTCTAGCACCATCAATG |
| RS14930-R | ATCCGCAGGCGACGTTTAAAAAAG |
| RS15110-F | GCGTCACTTGCCAAATGCCTT |
| RS15110-R | TACGATCTGACGCGTGAGGGT |
| RS15290-F | ACGCAGAAAAAGGTTTTGGTTTCATCG |
| RS15290-R | TGCTTGGTCTCCACGTTGACC |
| RS14365-F | ACGCCCTTGACGATGCATTTCT |
| RS14365-R | TCATGCGCGATCAGTGTAGCG |
| RS05215-F | TCAAAGTCGTGTAGACAGCCAACTAATG |
| RS05215-R | AACGGATGAAGATTGGGAAAATATTGAAAAGT |
| RS02395-F | GTTGGACAATGCCGAAGCGTG |
| RS02395-R | ACAACATACCTGCCCAACCCG |
| RS01005-F | GAAAATGAAGACAAGCGCCAAGCATT |
| RS01005-R | GTAGGCTGTTGGCCAAGGTGT |
| RS13885-F | TCACTGCGTTTAGCTTGTCCAATATGAT |
| RS13885-R | GAAAAGGTGCAAGCAGCTCGTC |
| RS08110-F | AGCAATAATGCCCCCGTGGAA |
| RS08110-R | GCGATGATTGGTTATCATCCCGATTATGAA |
| hu-F | TGCAGTTGCAGAGCAAGCTGA |
| hu-R | ACCAGCTTTGAATGCTGGAACTTTACT |
| pta-F | ACCTGAAGGAGAGGACGAACGT |
| pta-R | CTTGTGCTTGTTCTTCAGTCGCTTTAC |
| tpi-F | GATAATGGTGCGTTCACAGGTGAAAC |
| tpi-R | ACCACTTTCACGCTCTTCGTCTG |

Table S3 differentially down-regulated expressed genes in heme deficient strains

| Gene ID | Delta_hemA  /USA500  **(log_2_)** | Delta_hemB  /USA500  **(log_2_)** | Compli_hemA/  Delta_hemA  **(log_2_)** | Compli_hemB/  Delta_hemB  **(log_2_)** | Gene name | product | Pathway/function |
| --- | --- | --- | --- | --- | --- | --- | --- |
| USA300HOU_0008 | -1.92 | -2.14 | 2.30 | 1.20 | hutH | histidine ammonia-lyase | Histidine metabolism |
| USA300HOU_0181 | -3.00 | -2.90 | 4.10 | 1.52 | aldH2 | aldehyde dehydrogenase | Multiple metabolism |
| USA300HOU_0182 | -4.38 | -3.40 | 2.24 | 3.09 | / | cation efflux family protein | Transporters |
| USA300HOU_0205 | -9.07 | -6.34 | 4.93 | 5.41 | / | N-acetylmuramic acid 6-phosphate etherase | Amino sugar and nucleotide sugar metabolism |
| USA300HOU_0206 | -7.25 | -5.27 | 3.69 | 4.57 | / | permease | Starch and sucrose metabolism |
| USA300HOU_0207 | -6.20 | -4.25 | 2.93 | 3.39 | / | RpiR family transcriptional regulator | murPQ operon repressor |
| USA300HOU_0229 | -3.29 | -2.96 | 2.58 | 2.45 | / | sugar phosphate transport protein | Transporters |
| USA300HOU_0357 | -3.50 | -3.91 | 2.14 | 2.58 | glpT | glycerol-3-phosphate transporter | Transporters |
| USA300HOU_0642 | -6.06 | -4.89 | 5.08 | 4.46 | / | recombinase | Other chromosome partitioning proteins |
| USA300HOU_0643 | -5.71 | -3.71 | 4.09 | 2.83 | mnhA1 | Na+:H+ antiporter subunit A | Transporters |
| USA300HOU_0644 | -5.06 | -2.89 | 3.51 | 2.51 | mnhB1 | Na+:H+ antiporter subunit B | Transporters |
| USA300HOU_0645 | -3.82 | -2.23 | 3.00 | 1.48 | mnhC1 | Na+:H+ antiporter subunit C | Transporters |
| USA300HOU_0646 | -4.28 | -2.34 | 2.68 | 1.50 | mnhD1 | Na+:H+ antiporter subunit D | Transporters |
| USA300HOU_0647 | -4.24 | -1.87 | 2.39 | 1.28 | mnhE1 | Na+:H+ antiporter subunit E | Transporters |
| USA300HOU_0846 | -4.41 | -1.99 | 2.17 | 1.31 | / | thiol reductase thioredoxin | / |
| USA300HOU_0916 | -2.50 | -2.44 | 3.06 | 1.80 | rocD | ornithine--oxo-acid transaminase | Arginine and proline metabolism |
| USA300HOU_0917 | -1.85 | -2.24 | 2.34 | 1.49 | gudB | NAD-specific glutamate dehydrogenase | Alanine, aspartate and glutamate metabolism |
| USA300HOU_0919 | -4.49 | -4.96 | 3.39 | 4.81 | argH | argininosuccinate lyase | Alanine, aspartate and glutamate metabolism |
| USA300HOU_0920 | -4.56 | -5.03 | 3.16 | 4.91 | argG | argininosuccinate synthase | Alanine, aspartate and glutamate metabolism |
| USA300HOU_1009 | -3.23 | -2.99 | 2.78 | 2.88 | purE | phosphoribosylaminoimidazole carboxylase, catalytic subunit | Purine metabolism |
| USA300HOU_1010 | -2.98 | -3.43 | 2.73 | 3.07 | purK | N5-carboxyaminoimidazole ribonucleotide synthase | Purine metabolism |
| USA300HOU_1014 | -5.09 | -4.89 | 4.45 | 4.18 | purL | phosphoribosylformylglycinamidine synthase subunit PurL | Purine metabolism |
| USA300HOU_1015 | -4.92 | -5.36 | 4.42 | 4.55 | purF | amidophosphoribosyltransferase | Purine metabolism |
| USA300HOU_1016 | -4.97 | -5.19 | 4.50 | 4.40 | purM | phosphoribosylformylglycinamidine cyclo-ligase | Purine metabolism |
| USA300HOU_1017 | -3.82 | -4.23 | 3.51 | 3.45 | purH | bifunctional phosphoribosylaminoimidazolecarboxamide formyltransferase | Purine metabolism |
| USA300HOU_1018 | -3.23 | -3.95 | 3.02 | 3.00 | purD | phosphoribosylamine--glycine ligase | Purine metabolism |
| USA300HOU_1230 | -3.19 | -2.96 | 1.93 | 1.66 | glpF | glycerol transporter | Transporters |
| USA300HOU_1409 | -1.78 | -1.83 | 1.82 | 1.83 | / | heptaprenyl pyrophosphate synthase subunit A | menaquinone biosynthesis |
| USA300HOU_1605 | -4.93 | -3.32 | 3.37 | 1.77 | / | YcsF family protein | carbohydrate metabolism |
| USA300HOU_1606 | -4.85 | -3.02 | 3.63 | 1.85 | / | acetyl-CoA carboxylase biotin carboxylase subunit | Fatty acid biosynthesis |
| USA300HOU_1608 | -4.25 | -2.84 | 2.54 | 1.41 | / | allophanate hydrolase | Urine metabolism |
| USA300HOU_1674 | -3.10 | -4.20 | 3.47 | 3.45 | gapB | glyceraldehyde-3-phosphate dehydrogenase | Multiple metabolism |
| USA300HOU_1696 | -4.10 | -3.23 | 2.75 | 2.25 | ald | alanine dehydrogenase | Alanine, aspartate and glutamate metabolism |
| USA300HOU_1722 | -2.92 | -2.73 | 3.90 | 2.52 | acsA1 | acetate--CoA ligase | Glycolysis / Gluconeogenesis |
| USA300HOU_1723 | -2.36 | -2.72 | 1.83 | 1.86 | acuA | acetoin dehydrogenase | acetoin catabolic process |
| USA300HOU_1724 | -2.92 | -3.09 | 2.59 | 1.93 | acuC | acetoin utilization protein | acetoin catabolic process |
| USA300HOU_1756 | -3.85 | -2.94 | 4.20 | 2.14 | putA | proline dehydrogenase | Arginine and proline metabolism |
| USA300HOU_1758 | -2.28 | -2.63 | 2.27 | 1.41 | ribA | riboflavin biosynthesis protein RibBA | Riboflavin metabolism |
| USA300HOU_1759 | -2.31 | -2.42 | 1.96 | 1.14 | ribB | riboflavin synthase subunit alpha | Riboflavin metabolism |
| USA300HOU_1778 | -3.02 | -3.49 | 3.13 | 1.97 | pckA | phosphoenolpyruvate carboxykinase (ATP) | Glycolysis / Gluconeogenesis |
| USA300HOU_1808 | -3.23 | -1.93 | 1.30 | 1.45 | epiG | lantibiotic ABC transporter protein | Quorum sensing |
| USA300HOU_1809 | -4.16 | -2.37 | 2.04 | 2.04 | epiE | lantibiotic transport system permease protein | Quorum sensing |
| USA300HOU_1810 | -5.10 | -3.11 | 2.72 | 2.58 | epiF | lantibiotic transport system ATP-binding protein | Quorum sensing |
| USA300HOU_1879 | -3.07 | -1.61 | 1.41 | 1.32 | / | Ribonuclease BN | hydrolase |
| USA300HOU_1891 | -1.89 | -2.86 | 2.16 | 2.59 | / | non-heme ferritin | cellular iron ion homeostasis |
| USA300HOU_1918 | -3.00 | -1.97 | 1.95 | 1.08 | / | sodium-dependent dicarboxylate transporter SdcS | Transporters |
| USA300HOU_2031 | -16.87 | -4.30 | #N/A | 2.76 | hld | delta-hemolysin | Quorum sensing |
| USA300HOU_2154 | -2.03 | -1.98 | 2.69 | 1.04 | arg | arginase | Arginine biosynthesis |
| USA300HOU_2175 | -4.62 | -2.16 | 3.22 | 1.59 | asp23 | alkaline shock protein 23 | Transcription factor |
| USA300HOU_2178 | -5.64 | -2.49 | 2.87 | 1.62 | / | betaine ABC transporter permease | Transporters |
| USA300HOU_2200 | -6.87 | -2.79 | 3.40 | 2.11 | / | toxin |  |
| USA300HOU_2310 | -4.41 | -4.60 | 4.32 | 3.89 | hutI | imidazolonepropionase | Histidine metabolism |
| USA300HOU_2311 | -5.36 | -5.04 | 5.05 | 4.05 | hutU | urocanate hydratase | Histidine metabolism |
| USA300HOU_2314 | -3.45 | -1.88 | 2.27 | 1.47 | hutG | formimidoylglutamase | Histidine metabolism |
| USA300HOU_2424 | -4.85 | -4.05 | 4.66 | 2.90 | / | alanine glycine permease | Transporters |
| USA300HOU_2499 | -4.06 | -3.19 | 3.31 | 2.34 | / | glucarate transporter | Transporters |
| USA300HOU_2549 | -2.43 | -2.66 | 2.80 | 1.61 | / | 1-pyrroline-5-carboxylate dehydrogenase | Alanine, aspartate and glutamate metabolism |
| USA300HOU_2557 | -3.20 | -2.14 | 1.21 | 1.66 | crtM | dehydrosqualene synthase | Carotenoid biosynthesis |
| USA300HOU_2558 | -4.51 | -2.76 | 2.19 | 1.98 | / | glycosyl transferase, group 2 family protein | Carotenoid biosynthesis |
| USA300HOU_2559 | -5.58 | -3.48 | 3.36 | 2.92 | / | diapolycopene oxygenase | Carotenoid biosynthesis |
| USA300HOU_2560 | -7.52 | -4.18 | 5.16 | 4.09 | / | glycosyl-4,4'-diaponeurosporenoate acyltransferase | Carotenoid biosynthesis |
| USA300HOU_2610 | -3.99 | -3.31 | 2.47 | 2.83 | betT | choline transporter BetT | Transporters |
| USA300HOU_2655 | -2.14 | -3.19 | 1.79 | 2.26 | / | flavin reductase | oxidation-reduction process |
| USA300HOU_2671 | -7.04 | -4.32 | 3.15 | 3.28 | lip | lipase | Lipid metabolism |

Table S4 differentially up-regulated expressed genes in heme deficient strains

| Gene ID | Delta_hemA  /USA500  **(log_2_)** | Delta_hemB  /USA500  **(log_2_)** | Compli_hemA/  Delta_hemA  **(log_2_)** | Compli_hemB/  Delta_hemB  **(log_2_)** | Gene name | product | Pathway/function |
| --- | --- | --- | --- | --- | --- | --- | --- |
| USA300HOU_0226 | 2.42 | 1.72 | -1.46 | -2.39 | / | xylose isomerase | Base excision repair |
| USA300HOU_0249 | 3.06 | 2.05 | -1.38 | -2.91 | hmp | nitric oxide dioxygenase | Salmonella infection |
| USA300HOU_0396 | 3.47 | 1.80 | -2.83 | -2.05 | / | hypothetical protein ERS140147_02613 |  |
| USA300HOU_0457 | 2.32 | 1.83 | -2.62 | -3.04 | / | NADH dehydrogenase subunit 5 | Oxidative phosphorylation |
| USA300HOU_0458 | 2.66 | 2.00 | -2.40 | -2.81 | / | UPF0753 protein | / |
| USA300HOU_0762 | 3.43 | 2.41 | -4.65 | -1.37 | / | iron complex transport system substrate-binding protein, partial | ABC transporters |
| USA300HOU_0803 | 2.02 | 1.67 | -1.51 | -1.82 | pgk | phosphoglycerate kinase | Glycolysis |
| USA300HOU_0804 | 1.99 | 1.59 | -1.56 | -1.75 | tpi | triose-phosphate isomerase | Glycolysis |
| USA300HOU_0805 | 2.03 | 1.60 | -1.68 | -1.96 | pgm | 2,3-bisphosphoglycerate-independent phosphoglycerate mutase | Glycolysis |
| USA300HOU_0897 | 2.18 | 1.63 | -1.57 | -2.21 | / | iron-sulfur cluster assembly protein | iron-sulfur cluster binding |
| USA300HOU_1417 | 2.03 | 1.92 | -1.54 | -3.01 | / | thioredoxin reductase | Metabolism of other amino acids/thioredoxin reductase |
| USA300HOU_2128 | 3.92 | 2.86 | -2.00 | -1.87 | dps | DNA starvation/stationary phase protection protein | starvation-inducible DNA-binding protein |
| USA300HOU_2201 | 4.82 | 4.20 | -2.38 | -3.13 | alsD | alpha-acetolactate decarboxylase | Butanoate metabolism |
| USA300HOU_2202 | 4.25 | 4.09 | -1.97 | -3.28 | alsS | acetolactate synthase | Butanoate metabolism |
| USA300HOU_2333 | 2.64 | 2.35 | -1.45 | -3.35 | / | multidrug MFS transporter | Multidrug resistance, efflux pump EmrAB |
| USA300HOU_2334 | 3.05 | 2.54 | -1.74 | -3.07 | / | multidrug efflux protein | Multidrug resistance, efflux pump EmrAB |
| USA300HOU_2388 | 3.14 | 1.98 | -1.84 | -1.45 | / | antitoxin YefM | antitoxin YefM |
| USA300HOU_2439 | 2.25 | 2.17 | -1.30 | -2.71 | / | YdeI | YdeI |
| USA300HOU_2526 | 2.96 | 2.68 | -1.04 | -3.13 | / | thiol reductase thioredoxin | glycerol ether metabolic process |
| USA300HOU_2599 | 3.08 | 1.80 | -1.89 | -2.46 | fdaB | class I fructose-bisphosphate aldolase | Glycolysis |
| USA300HOU_2633 | 3.69 | 5.85 | -1.21 | -6.29 | arcD3 | APC family amino acid-polyamine-organocation transporter | arginine:ornithine antiporter / lysine permease |
| USA300HOU_2634 | 4.03 | 5.77 | -1.40 | -5.59 | arcB3 | ornithine carbamoyltransferase | Arginine biosynthesis |
| USA300HOU_2635 | 4.47 | 5.98 | -1.79 | -5.74 | arcA3 | arginine deiminase | Arginine biosynthesis |

Table S5 Quantitive Realtime PCR fold change of heme deficient strains

|  | Quantitive Realtime PCR fold change（**log_2_**） | | | | RNA-seq fold change（**log_2_**） | | | |
| --- | --- | --- | --- | --- | --- | --- | --- | --- |
| Gene name | Delta_hemA/  USA500 | Delta_hemB/  USA500 | Compli_hemA/  Delta_hemA | Compli_hemB/  Delta_hemB | Delta_hemA/  USA500 | Delta_hemB/  USA500 | Compli_hemA/  Delta_hemA | Compli_hemB/  Delta_hemB |
| **USA300HOU_0204** | -6.32 | -4.33 | 4 | 2.8 | -8.32 | -5.60 | 4.46 | 4.87 |
| mnhA1 | -3.28 | -2.01 | 1.28 | -0.25 | -5.61 | -3.67 | 4.00 | 2.88 |
| **mnhB1** | -3.75 | -5.00 | 1.1 | 1.42 | -4.96 | -2.85 | 3.43 | 2.56 |
| **mnhD1** | -4.03 | -4.78 | 1.4 | 1.12 | -4.18 | -2.30 | 2.59 | 1.55 |
| argH | -2.52 | -2.51 | 3.56 | -3.56 | -4.46 | -4.99 | 3.08 | 4.96 |
| **purQ** | -4.41 | -3.19 | 3.27 | 2.67 | -4.13 | -6.62 | 3.76 | 6.34 |
| **purL** | -2.52 | -2.55 | 3.02 | 0.42 | -4.99 | -4.84 | 4.36 | 4.24 |
| purF | -2.51 | 5.23 | 4.3 | -7.96 | -4.81 | -5.32 | 4.33 | 4.60 |
| **purM** | -5.42 | -4.20 | 5.42 | 2.21 | -4.86 | -5.15 | 4.41 | 4.45 |
| **USA300HOU**_**1606** | -5.64 | -4.13 | 4.54 | 2.36 | -4.74 | -2.98 | 3.54 | 1.90 |
| **epiF** | -4.58 | -2.57 | 4.01 | 0.36 | -5.00 | -3.07 | 2.64 | 2.63 |
| **asp23** | -5.23 | -2.78 | 4.49 | 1.19 | -4.52 | -2.12 | 3.13 | 1.64 |
| **USA300HOU_2178** | -5.27 | -3.48 | 4.07 | 1.52 | -5.54 | -2.45 | 2.78 | 1.67 |
| alsD | -1.11 | -0.27 | -0.17 | -3.46 | 4.92 | 4.24 | -2.46 | -3.08 |
| alsS | -0.72 | 0.36 | -1.75 | -7.55 | 4.36 | 4.13 | -2.06 | -3.23 |
| hutI | 0.20 | 0.37 | 2.6 | 0.05 | -4.31 | -4.55 | 4.23 | 3.94 |
| **hutU** | -5.50 | -3.61 | 4.59 | 2.83 | -5.25 | -5.00 | 4.96 | 4.10 |
| htrA | -2.53 | -0.75 | -1.8 | 3.84 | -3.09 | -4.44 | 2.28 | 5.96 |
| **USA300HOU_2424** | -2.09 | -1.13 | 3.34 | 0.04 | -4.74 | -4.01 | 4.57 | 2.95 |
| USA300HOU_2561 | -2.38 | -0.31  -2.24 | -1.52 | -5.38 | -5.30 | -6.50 | 3.87 | 4.61 |
| lip | -4.80 |  | 0.4 | -2.14 | -6.96 | -4.28 | 3.08 | 3.32 |
| **USA300HOU_2526** | 1.08 | 0.45 | -0.02 | -1.95 | 3.05 | 2.72 | -1.11 | -3.08 |
| **ydeI** | 4.41 | 4.53 | -3.35 | -8.45 | 2.35 | 1.76 | -1.38 | -2.66 |
| hmp | 1.86 | 0.91 | 0.11 | -2.56 | 3.17 | 2.09 | -1.47 | -2.86 |
| USA300HOU_0762 | 1.74 | 0.99 | -0.65 | 0.38 | 3.53 | 2.45 | -4.74 | -1.32 |
| **USA300HOU_0822** | 1.24 | 0.46 | -0.47 | -1.03 | 4.86 | 3.49 | -2.83 | -1.02 |
| **dps** | 2.20 | 1.55 | -0.47 | -6.61 | 4.02 | 2.90 | -2.08 | -1.83 |
| **USA300HOU_2333** | 1.85 | 1.18 | -0.63 | -2.87 | 2.75 | 2.39 | -1.54 | -3.30 |
| **USA300HOU_2334** | 2.41 | 1.68 | -4.91 | -7.43 | 3.15 | 2.58 | -1.83 | -3.02 |
| USA300HOU_2388 | 0.67 | 0.37 | 0.66 | -1.77 | 3.22 | 2.01 | -1.91 | -1.41 |
| **arcD3** | 4.18 | 4.75 | -5.95 | -7.87 | 3.79 | 5.89 | -1.30 | -6.23 |
| **arcB3** | 2.26 | 2.61 | -0.9 | -4.35 | 4.14 | 5.81 | -1.49 | -5.54 |
| **arcA3** | 4.64 | 6.78 | -5.48 | -9.75 | 4.58 | 6.02 | -1.88 | -5.69 |

The gene names in bold showed the transcription fold change for the genes tested in RNA-seq had been confirmed in Quantitative Real-time PCR（-∆∆CT）, which were mean results for three tests.
