# Supplementary figures and images for: Involvement of Small Colony Variant-Related Heme Biosynthesis Genes in Staphylococcus aureus Persister Formation in vitro
Source: Front Microbiol. 2021 Dec 23;12:756809. doi: 10.3389/fmicb.2021.756809 (PMC8733728; doi:10.3389/fmicb.2021.756809)

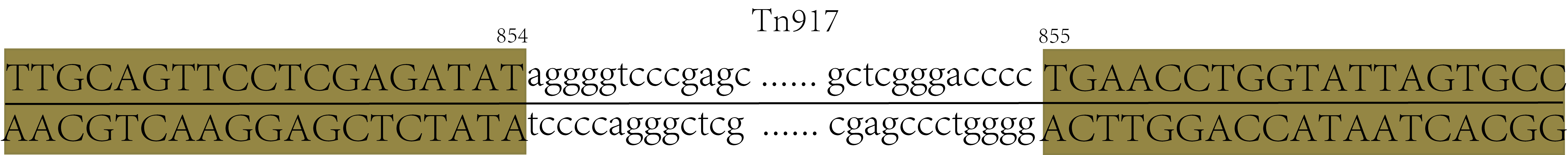

Supplement: Supplementary Figure S1 — Transposon insertion site in hemA. This figure indicates screened clones with Tn917 transposon insertion between nucleotides 854 and 855 of hemA. [file image_1.tif]
